# Copula-Based Uncertainty Quantification (Copula-UQ) for Multi-Sensor Data in Structural Health Monitoring

**DOI:** 10.3390/s20195692

**Published:** 2020-10-06

**Authors:** He-Qing Mu, Han-Teng Liu, Ji-Hui Shen

**Affiliations:** 1 School of Civil Engineering and Transportation, South China University of Technology, Guangzhou 510640, China; 201820106919@mail.scut.edu.cn (H.-T.L.); 201920106856@mail.scut.edu.cn (J.-H.S); 2 State Key Laboratory of Subtropical Building Science, South China University of Technology, Guangzhou 510640, China; 3 Key Laboratory of Earthquake Engineering and Engineering Vibration, Institute of Engineering Mechanics, China Earthquake Administration, Harbin 150080, China

**Keywords:** copula, missing data, multivariate random variable, probability density distribution, structural health monitoring

## Abstract

The problem of uncertainty quantification (UQ) for multi-sensor data is one of the main concerns in structural health monitoring (SHM). One important task is multivariate joint probability density function (PDF) modelling. Copula-based statistical inference has attracted significant attention due to the fact that it decouples inferences on the univariate marginal PDF of each random variable and the statistical dependence structure (called copula) among the random variables. This paper proposes the Copula-UQ, composing multivariate joint PDF modelling, inference on model class selection and parameter identification, and probabilistic prediction using incomplete information, for multi-sensor data measured from a SHM system. Multivariate joint PDF is modeled based on the univariate marginal PDFs and the copula. Inference is made by combing the idea of the inference functions for margins and the maximum likelihood estimate. Prediction on the PDF of the target variable, using the complete (from normal sensors) or incomplete information (due to missing data caused by sensor fault issue) of the predictor variable, are made based on the multivariate joint PDF. One example using simulated data and one example using temperature data of a multi-sensor of a monitored bridge are presented to illustrate the capability of the Copula-UQ in joint PDF modelling and target variable prediction.

## 1. Introduction

The problem of uncertainty quantification (UQ) for multi-sensor data has been one of the main concerns in nondestructive testing and structural health monitoring (SHM) over the years [1,2,3,4,5,6,7,8,9]. One important task is multivariate joint probability density function (PDF) modelling. Due to irregularities of multi-sensor data, the joint PDF can be too complicated to be modelled by traditional approaches. For example, traditional multivariate PDFs (such as a multivariate normal distribution) cannot model the PDF with multiple peaks. The multivariate mixture PDFs (such as multivariate normal mixture model), utilized in SHM and damage detection [10,11,12,13], rely on the proper choice of the number and the type of the mixture distributions and an initial value of parameter vector in optimization [14]. The Nataf distribution, utilized in SHM and structural reliability [15,16], relies on the assumption that the transformed random variables, obtained from the marginal transformations of the original random variables, are multivariate normal distribution [17].

In recent years, copula-based statistical inference has attracted significant attention due to the fact that it decouples the inference on the univariate marginal PDF of each random variable and the statistics dependence structure (called copula) among the random variables. In the areas of the SHM and structural assessment, Zhang and Kim [18] investigated a way of detecting bridge damage for the long-term health monitoring by using the copula theory. Fan and Liu [19] predicted the dynamic reliability of a bridge system based on SHM data. Pan et al. [20] developed a copula-based approach to model the structural health of an operational metro tunnel in a dependent system. Liu et al. [21] considered the correlation between the fatigue equivalent stress and the stress cycle using the copula function in the fatigue reliability assessment. Srinivas et al. [22] proposed the multivariate simulation of dependent axle weights of different vehicle classes. Zhang et al. [23] investigated the specification of long-term design loads for offshore structures considering multiple environmental factors. 

Although the copula-based statistical inference has been widely applied, there are two limitations in previous works related to the SHM and structural assessment. The first limitation is insufficient types of probabilistic model candidates for univariate marginal PDF modelling. From the parametrization point of view, there are parametric models and nonparametric models for PDF modelling. The former type, assuming that sample data come from a distribution that has a fixed set of parameters, is suitable for data with regular statistical pattern; the latter type, being not specified a priori but being instead adaptively determined from data, is suitable for data with an irregular statistical pattern. In the SHM, it is well known that the statistical regularities of data from multiple sensors can be significantly different from each other. Thus, due to the complexity of real SHM data, only considering one type of probabilistic model in PDF modelling bounds the solution space for UQ, leading to incapability of capturing a statistical pattern of data. However, this important issue was not realized in previous works, so parametric models and nonparametric models were not considered simultaneously. For example, References [18,19,20,21,23] solely adopted parametric models, while Reference [22] solely adopted nonparametric models for univariate marginal PDF modelling. Thus, this paper attempts to break through this limitation by including sufficient types of probabilistic models as candidates. The second limitation is negligence of probabilistic prediction using available information, especially in the case of using the incomplete information of the predictor variable due to missing data caused by a sensor fault issue. For the works of the research area of SHM using the copula [18,19,20,21,22,23], it had not been realized that the joint PDF can be utilized for probabilistic prediction on the target variable using the available information of the predictor variable. Even for the very recent work of another research area using the copula [24], probabilistic prediction on the target variable is limited to the case using the complete information of the predictor variable only. However, the case of incomplete information of the predictor variable, due to missing data caused by a sensor fault issue, is critical and common in the SHM. Thus, this paper attempts to break through this limitation by conducting computation of marginalization and conditioning based on the copula-based joint PDF, for prediction on the PDF of the target variable using the complete (from normal sensors) or incomplete information (due to missing data caused by sensor fault issue) of the predictor variable.

This paper proposes the copula-based UQ (Copula-UQ), composing multivariate joint PDF modelling, inference on model class selection and parameter identification, and probabilistic prediction using incomplete information, for multi-sensor data measured from a SHM system. The proposed Copula-UQ contains two stages. The first stage is the copula-based multivariate joint PDF modelling. It is based on the univariate marginal PDFs and the copula. The second stage is copula-based inference and prediction. Inference, including determination of optimal parameters and selection of optimal model classes, is made by combining the idea of the inference functions for margins (IFM) and the maximum likelihood estimate (MLE). Prediction on the PDF of the target variable, using the complete or incomplete information of the predictor variable, are made based on the copula-based multivariate joint PDF.

The structure of this paper is outlined as follows. Section 2 presents copula-based multivariate joint PDF modelling, including model class candidates for univariate marginal PDFs and copula. Section 3 presents copula-based inference and prediction, including inference on univariate marginal PDFs and copula, and prediction on the target variable. Section 4 presents illustrative examples. One example using simulated data and one example using temperature data of multi-sensor of a monitored bridge are presented to illustrate the capability of the proposed Copula-UQ in joint PDF modelling and target variable prediction.

## 2. Copula-Based Multivariate Joint PDF Modelling

Let $p\left(x_{1},x_{2},\cdots ,x_{D}\right)$ denote the joint PDF of D random variables $\left(X_{1},X_{2},\ldots X_{D}\right)$, and $X\in {\mathbb{R}}^{D\times N}$ denote the measured data matrix with its component $X_{d,i}^{}$ being the d-th dimension of the i-th data point, with d=1,…,D and i=1,…,N. The copula-based multivariate joint PDF is to model $p\left(x_{1},x_{2},\cdots ,x_{D}\right)$ based on the univariate marginal PDFs $p\left(x_{d}\right),d=1,2,\ldots ,D$ of each random variable and the statistics dependence structure (called copula) among the random variables, given the measured data matrix X. 

### 2.1. Univariate Marginal PDFs

For the d-th univariate random variable $X_{d}$, consider a set of $N_{M}$ model class candidates, namely, $M_{d}^{m_{d}},\;m_{d}=1,2,\ldots ,N_{M}$. Each model class candidate represents a marginal PDF for $X_{d}$. It is obvious that a larger solution space for uncertainty quantification can be achieved with larger $N_{M}$. Let $p\left(x_{d}|\theta_{d}^{m_{d}},M_{d}^{m_{d}}\right)$ be the PDF of $x_{d}$ given the parameter vector $\theta_{d}^{m_{d}}\epsilon {\mathbb{R}}^{N\left(\theta_{d}^{m_{d}}\right)}$ of model class candidate $M_{d}^{m_{d}}$, where $N\left(\theta_{d}^{m_{d}}\right)$ is the number of parameters depending on $M_{d}^{m_{d}}$. As copula-based multivariate joint PDF modelling is flexible on the models for univariate marginal PDFs of different dimensions, a set of parametric and nonparametric models are introduced as model class candidates for selection of marginal PDFs. It is worth noting that the introduction of both parametric and nonparametric models simultaneously is necessary for breaking through the first limitation (i.e., insufficient types of probabilistic model candidates) described in Section 1 because it provides a large solution space for uncertainty quantification. This will be validated in Section 4–illustrative examples.

Parametric modeling methods assume that sample data come from a distribution that has a fixed set of parameters. In this paper, the parametric model class candidates include: Normal distribution, Lognormal distribution, Weibull distribution, Gamma distribution, Gumbel distribution and Uniform distribution.

Nonparametric models differ from parametric models in that the model structure is not specified a priori but is instead determined from data. The term nonparametric is not meant to imply that such models completely lack parameters but that the number and nature of the parameters are flexible and not fixed in advance. In this paper, the kernel density estimation method is used. Based on the d-th dimensional measured data $X_{d}={\left[X_{d,1}^{},X_{d,2}^{},\ldots ,X_{d,N}^{}\right]}^{T}$, the nonparametric kernel density function is adopted [25]:

(1) $$p\left(x_{d}|\theta_{d}^{m_{d}},M_{d}^{m_{d}}\right)=\frac{1}{N\theta_{d}^{m_{d}}}\overset{N}{\underset{j=1}{\sum }}K_{d}^{m_{d}}\left(\frac{x_{d}-X_{d,j}^{}}{\theta_{d}^{m_{d}}}\right)$$

where $\theta_{d}^{m_{d}}$ is the bandwidth ($\theta_{d}^{m_{d}}>0$) controlling the smoothness of the resulting probability density curve, and $K_{}^{m_{d}}\left(\cdot \right)$ is the kernel smoothing function depending on $M_{d}^{m_{d}}$ and $X_{d}$. Let $\varrho_{d,j}^{m_{d}}=\left(x_{d}-X_{d,j}^{}\right)/\theta_{d}^{m_{d}}$. Four kernel functions, including Normal kernel, Box kernel, Triangle kernel and Epanechnikov kernel, are introduced as follows:

(2) $$\mathrm{Normal}\;\mathrm{kernel}:\;K_{d}^{m_{d}}\left[\varrho_{d,j}^{m_{d}}\right]=\frac{1}{\;\sqrt{2\pi }}\exp\left\{-\frac{1}{2}{\left[\varrho_{d,j}^{m_{d}}\right]}^{2}\right\}$$

(3) $$\mathrm{Box}\;\mathrm{kernel}:\;K_{d}^{m_{d}}\left[\varrho_{d,j}^{m_{d}}\right]=\frac{1}{2}1_{\left|\varrho_{d,j}^{m_{d}}\right|\leq 1}\left[\varrho_{d,j}^{m_{d}}\right]$$

(4) $$\mathrm{Triangle}\;\mathrm{kernel}:\;K_{d}^{m_{d}}\left[\varrho_{d,j}^{m_{d}}\right]=\left[1-\varrho_{d,j}^{m_{d}}\right]1_{\left|\varrho \right|\leq 1}\left[\varrho_{d,j}^{m_{d}}\right]$$

(5) $$\mathrm{Epanechnikov}\;\mathrm{kernel}:K_{d}^{m_{d}}\left[\varrho_{d,j}^{m_{d}}\right]=\frac{3}{4}\left\{1-{\left[\varrho_{d,j}^{m_{d}}\right]}^{2}\right\}1_{\left|\varrho_{d,j}^{m_{d}}\right|\leq 1}\left[\varrho_{d,j}^{m_{d}}\right]$$

where $1_{\left|\varrho_{d,j}^{m_{d}}\right|\leq 1}\left[\varrho_{d,j}^{m_{d}}\right]$ is the indicator function.

### 2.2. Copula

Let $P\left(x_{d}|\theta_{d}^{m_{d}},M_{d}^{m_{d}}\right)$ denote the marginal cumulative distribution function (CDF) of $X_{d}$ based on $\theta_{d}^{m_{d}}$ and $M_{d}^{m_{d}}$. The probability $u_{d}^{m_{d}}$ of $X_{d}\leq x_{d}$ can be obtained as:

(6) $$u_{d}^{m_{d}}=P\left(x_{d}|\theta_{d}^{m_{d}},M_{d}^{m_{d}}\right)$$

Applying the probability integral transform to $X_{d}$, the univariate random variable $U_{d}^{m_{d}}$ can be obtained:

(7) $$U_{d}^{m_{d}}=P\left(X_{d}|\theta_{d}^{m_{d}},M_{d}^{m_{d}}\right)$$

where $U_{d}^{m_{d}}$ follows standard uniform distribution on the interval [0,1]. 

The copula of $\left(X_{1},X_{2},\ldots X_{D}\right)$ is defined as a joint CDF of $\left(U_{1}^{m_{1}},U_{2}^{m_{2}},\cdots ,U_{D}^{m_{D}}\right)$ [26]:

(8) $$C\left(u_{1}^{m_{1}},u_{2}^{m_{2}},\cdots ,u_{D}^{m_{D}}\right)=P\left(U_{1}^{m_{1}}\leq u_{1}^{m_{1}},U_{2}^{m_{2}}\leq u_{2}^{m_{2}},\ldots ,U_{D}^{m_{D}}\leq u_{D}^{m_{D}}\right)$$

That is $C:{\left[0,1\right]}^{D}\to \left[0,1\right]$ is a D-dimensional copula if C is a joint CDF of a D-dimensional random vector on the unit hypercube ${\left[0,1\right]}^{D}$, with the marginal PDF of each component of the random vector following the standard uniform PDF on the interval [0,1]. The copula C describes exactly the dependence structure among the random variables.

Consider the joint CDF of $\left(X_{1},X_{2},\ldots X_{D}\right)$:

(9) $$P\left(x_{1},\cdots ,x_{D}\right)=P\left(X_{1}\leq x_{1},\ldots ,X_{D}\leq x_{D}\right)$$

Sklar’s theorem states that there exists a D-dimensional copula, such that [27]:

(10) $$P\left(x_{1},\cdots ,x_{D}\right)=C\left(u_{1}^{m_{1}},\cdots ,u_{D}^{m_{D}}|\psi \right)=C\left(P(x_{1}|\theta_{1}^{m_{1}},M_{1}^{m_{1}}),\cdots ,P(x_{D}|\theta_{D}^{m_{D}},M_{D}^{m_{D}})|\psi \right)$$

where ψ is the parameter vector of the copula. If $P\left(x_{d}|\theta_{d}^{m_{d}},M_{d}^{m_{d}}\right)$ are continuous, the copula is unique; otherwise, it is uniquely determined on the Cartesian product of the ranges of the marginal CDFs. Sklar’s theorem clearly indicates that the joint CDF of random variables can be characterized by a copula in terms of the marginal CDFs. 

Thus, the joint PDF, $p\left(x_{1},x_{2},\cdots ,x_{D}\right),$ can be derived from its joint CDF, $P\left(x_{1},x_{2}\cdots ,x_{D}\right),$ of Equation (10):

(11) $$p\left(x_{1},x_{2},\cdots ,x_{D}\right)=c\left(u_{1}^{m_{1}},u_{2}^{m_{2}},\cdots ,u_{D}^{m_{D}}|\psi \right)\cdot \overset{D}{\underset{d=1}{\prod }}p(x_{d}|\theta_{d}^{m_{d}},M_{d}^{m_{d}})$$

where $c\left(u_{1}^{m_{1}},u_{2}^{m_{2}},\cdots ,u_{D}^{m_{D}}|\psi \right)$ is the copula density function:

(12) $$c\left(u_{1}^{m_{1}},u_{2}^{m_{2}},\cdots ,u_{D}^{m_{D}}|\psi \right)=\frac{\partial^{D}C\left(u_{1}^{m_{1}},u_{2}^{m_{2}},\cdots ,u_{D}^{m_{D}}|\psi \right)}{\partial u_{1}^{m_{1}}\partial u_{2}^{m_{2}}\cdots \partial u_{D}^{m_{D}}}$$

and $p(x_{d}|\theta_{d}^{m_{d}},M_{d}^{m_{d}})$ is the marginal PDF of $X_{d}$.

In this paper, the multivariate Gaussian copula and the associated copula density function are introduced as follows [28]:

(13) $$C\left(u_{1}^{m_{1}},u_{2}^{m_{2}},\cdots ,u_{D}^{m_{D}}|\psi \right)=\Phi_{\rho \left(\zeta \right)}\left(\zeta \right)$$

(14) $$c\left(u_{1}^{m_{1}},u_{2}^{m_{2}},\cdots ,u_{D}^{m_{D}}|\psi \right)={\left|\rho \left(\zeta \right)\right|}^{-\frac{1}{2}}\exp\left\{-\frac{1}{2}\zeta_{}^{T}\left[\rho {\left(\zeta \right)}^{-1}-I\right]\zeta \right\}$$

(15) $$\zeta ={\left[\Phi^{-1}\left(u_{1}^{m_{1}}\right),\Phi^{-1}\left(u_{2}^{m_{2}}\right),\cdots ,\Phi^{-1}\left(u_{D}^{m_{D}}\right)\right]}^{T}$$

where $\Phi^{-1}\left(\cdot \right)$ is the inverse CDF of the univariate standard normal distribution function, $\Phi_{\rho \left(\zeta \right)}\left(\cdot \right)$ is the joint CDF of a D-dimensional normal distribution with mean vector zero and covariance matrix equal to the correlation coefficient matrix $\rho \left(\zeta \right)$ of ζ defined in Equation (15), the parameter vector ψ is the collection of the off-diagonal elements of the upper triangular part of $\rho \left(\zeta \right)$ and I is a D-dimensional identity matrix.

## 3. Copula-Based Inference and Prediction

### 3.1. Inference on Univariate Marginal PDFs and Copula

This stage is to make inference on $\Theta =\left\{\theta_{1}^{m_{1}},\ldots \theta_{D}^{m_{D}}\right\}$ (parameters of the marginal PDFs), $Μ=\left\{M_{1}^{m_{1}},\ldots ,M_{D}^{m_{D}}\right\}$ (model class candidates of the marginal PDFs) and ψ (parameters of the multivariate Gaussian-copula), based on the measured data matrix $X\in {\mathbb{R}}^{D\times N}$ and the probability matrix $U\in {\mathbb{R}}^{D\times N}$, with its component $U_{d,i}^{}=P\left(X_{d,i}^{}|\Theta ,Μ\right)$. Under the idea of the inference functions for margins (IFM) [29], Θ (along with Μ) and ψ can be determined separately. 

For the univariate marginals, the optimal parameter values can be obtained by the MLE:

(16) $${\hat{\theta }}_{d}^{m_{d}}=\underset{\theta_{d}^{m_{d}}}{\mathrm{argmax}}\log\left\{Lp\left(X_{d}|\theta_{d}^{m_{d}},M_{d}^{m_{d}}\right)\right\},\;d=1,\ldots ,D$$

(17) $$Lp\left(X_{d}|\theta_{d}^{m_{d}},M_{d}^{m_{d}}\right)=\overset{N}{\underset{i=1}{\prod }}\left[p\left(X_{d,i}^{}|\theta_{d}^{m_{d}},M_{d}^{m_{d}}\right)\right]$$

where $\log\left\{\cdot \right\}$ is the logarithmic function. For most of the parametric models, analytical forms for the optimal parameters can be derived (for example, see Reference [30]). For nonparametric models, the optimal value, ${\hat{\theta }}_{d}^{m_{d}}$ (bandwidth), can be obtained by considering the asymptotic mean integrated squared error solution [31]:

(18) $${\hat{\theta }}_{d}^{m_{d}}={\left(\frac{4{\hat{\sigma }}_{d}^{5}}{3N}\right)}^{-\frac{1}{5}}$$

(19) $${\hat{\sigma }}_{d}^{}=\frac{Q_{d}\left(0.75\right)-Q_{d}\left(0.25\right)}{1.349}$$

where $Q_{d}\left(0.75\right)$ and $Q_{d}\left(0.25\right)$ are the 75% and 25% quantiles of $X_{d}$. 

The optimal marginal PDFs ($\hat{Μ}=\left\{{\hat{M}}_{1}^{},\ldots ,{\hat{M}}_{D}^{}\right\}$) are selected by comparing the optimal likelihood values of different $m_{d}$:

(20) $${\hat{M}}_{d}\;=\;\arg\underset{M_{d}^{m_{d}}}{\max}Lp\left(X_{d}|{\hat{\theta }}_{d}^{m_{d}},M_{d}^{m_{d}}\right),\;m_{d}=1,\cdots ,N_{M},d=1,\ldots ,D$$

After selecting $\hat{Μ}$, the optimal parameters associated with $\hat{Μ}$ are denoted as $\hat{\Theta }=\left\{{\hat{\theta }}_{1}^{},\ldots {\hat{\theta }}_{D}^{}\right\}$. Based on $\hat{Μ}$ and $\hat{\Theta }$, the component of the optimal probability matrix ${\hat{U}}_{d,i}^{}=P\left(X_{d,i}^{}|\hat{\Theta },\hat{Μ}\right)$ can be obtained. The optimal values of the parameters of the multivariate Gaussian-copula $\hat{\psi }$ can be determined by considering the optimization on $Lc\left(\hat{U}|\psi \right)$:

(21) $$\hat{\psi }=\underset{\psi }{\mathrm{argmax}}\;\log\left\{Lc\left(\hat{U}|\psi \right)\right\}$$

(22) $$Lc\left(\hat{U}|\psi \right)=\overset{N}{\underset{i=1}{\prod }}\left[c\left({\hat{U}}_{1,i}^{},{\hat{U}}_{2,i}^{},\cdots ,{\hat{U}}_{D,i}^{}|\psi \right)\right]$$

where $c\left({\hat{U}}_{1,i}^{},{\hat{U}}_{2,i}^{},\cdots ,{\hat{U}}_{D,i}^{}|\psi \right)$ is obtained by substituting ${\hat{U}}_{1,i}^{},{\hat{U}}_{2,i}^{},\cdots ,{\hat{U}}_{D,i}^{}$ into Equation (14). For the multivariate Gaussian copula, the optimal parameter $\hat{\psi }$ is the collection of the off-diagonal elements of the upper triangular part of $\rho \left(\zeta \right)$, with each component being Pearson’s correlation coefficient.

### 3.2. Prediction on the Target Variable Given Complete or Incomplete Information (Due to Missing Data Caused by a Sensor Fault Issue)

Let $p\left(x_{1},x_{2},\cdots ,x_{D}|\hat{\psi },\hat{\Theta },\hat{Μ}\right)$ denote the multivariate joint PDF obtained by substituting $\hat{\psi },\hat{\Theta },\hat{Μ}$ into Equation (11). Let the target variable be the set containing the selected components of $X_{1},X_{2},\ldots X_{D}$ for prediction, and the predictor variable be the complement of the target variable. As the joint PDF contains all the statistical information about the random variables $\left(X_{1},X_{2},\ldots X_{D}\right)$, prediction on the PDF of the target variable can be obtained using the complete or incomplete information of the predictor variable. Let $x_{\mathrm{ta}}$, $x_{o}$ and $x_{\mathrm{uo}}$ denote the target variable, observed predictor variable and unobserved predictor variable, respectively. It is worth noting that the existence of unobserved predictor variable $x_{\mathrm{uo}}$ is very common in the SHM as it represents missing data of the corresponding channels of fault sensors. However, the very recent work of copula-based prediction [24] was still incapable of tackling the existence of $x_{\mathrm{uo}}$ in its prediction phase. Here, by conducting computation of marginalization and conditioning on the copula-based multivariate joint PDF, the prediction on the PDF of $x_{\mathrm{ta}}$ based on the observation $x_{o}={\tilde{x}}_{o}$ only (that is, available information only) can be obtained by:

(23) $$p\left(x_{\mathrm{ta}}|x_{o}={\tilde{x}}_{o}\right)=\frac{p\left(x_{\mathrm{ta}},x_{o}={\tilde{x}}_{o}\right)}{p\left(x_{o}={\tilde{x}}_{o}\right)}=\frac{\int {\left.p\left(x_{1},x_{2},\cdots ,x_{D}|\hat{\psi },\hat{\Theta },\hat{Μ}\right)\right|}_{x_{o}={\tilde{x}}_{o}}dx_{\mathrm{uo}}}{\iint {\left.p\left(x_{1},x_{2},\cdots ,x_{D}|\hat{\psi },\hat{\Theta },\hat{Μ}\right)\right|}_{x_{o}={\tilde{x}}_{o}}dx_{\mathrm{uo}}dx_{\mathrm{ta}}}$$

where ${\left.p\left(x_{1},x_{2},\cdots ,x_{D}|\hat{\psi },\hat{\Theta },\hat{Μ}\right)\right|}_{x_{o}={\tilde{x}}_{o}}$ is the copula-based multivariate joint PDF with substituting $x_{o}={\tilde{x}}_{o}$. The predicted PDF $p\left(x_{\mathrm{ta}}|x_{o}={\tilde{x}}_{o}\right)$ contains all the statistical information about $x_{\mathrm{ta}}$ based on the information $x_{o}={\tilde{x}}_{o}$. Accordingly, the predicted value (mean) and the associated uncertainty (standard deviation) of $x_{\mathrm{ta}}$ can be obtained.

## 4. Illustrative Examples

One example of simulation data and one example of real SHM data are demonstrated. For the simulation data example, the design of it is to validate the following three critical issues: (1) the necessary introduction of both parametric and nonparametric models for breaking through the first limitation (i.e., insufficient types of probabilistic model candidates), (2) the capability of the multivariate joint PDF modelling of the proposed Copula-UQ (as the true joint PDF is known) and (3) the performance of the proposed Equation (23) for prediction on the target variable given complete or incomplete information (due to missing data caused by a sensor fault issue). For the real SHM data example, the performance of the proposed Copula-UQ for prediction on the target variable under complete (normal sensors) or incomplete (fault sensors) information is further validated by considering the following two cases: (1) the test dataset is identical to the training dataset, and (2) the test dataset is different from the training dataset.

### 4.1. Simulation Data

This example applies the proposed Copula-UQ for multivariate joint PDF modelling and prediction of five-dimensional random variables, $X={\left[X_{1},\ldots ,X_{5}\right]}^{T}$. First, five uncorrelated random variables, $Z={\left[Z_{1},\ldots ,Z_{5}\right]}^{T}$, with different marginal PDFs are constructed (see Table 1). Then, the random variables $X={\left[X_{1},\ldots ,X_{5}\right]}^{T}$ are obtained by applying an affine transformation X=AZ with A given as:

(24) $$A=\left[\begin{array}{ccccc} 0.5 & 0.2 & 0 & 0.2 & 0.1\\ 0 & 0.6 & 0 & 0.2 & 0.2\\ 0 & 0 & 1 & 0 & 0\\ 0.3 & 0 & 0 & 0.7 & 0\\ 0 & 0.3 & 0 & 0 & 0.7 \end{array}\right]$$

**Figure 1:**
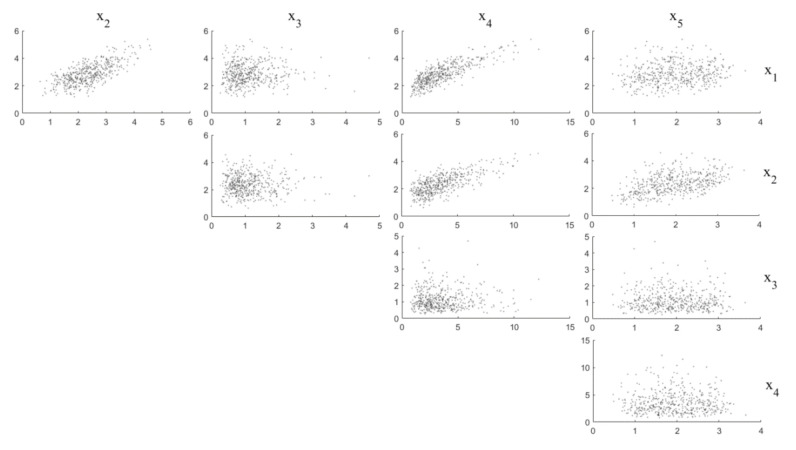
Scatter plot of the simulated data of $X_{1}$ to $X_{5}$ (*N* = 500).

Thus, the analytical form of the joint PDF of X is:

(25) $$p_{X}\left(x\right)=\frac{p_{Z}\left(z=A^{-1}x\right)}{\det\left|A\right|}$$

where $p_{Z}\left(z=A^{-1}x\right)$ is the joint PDF of Z with $z=A^{-1}x$. Figure 1 shows the scatter plot of the simulated data for $X_{1}$ to $X_{5}$ (N=500). The correlation coefficient matrix for X is:

(26) $$corr\left(X,X\right)\;=\;\left[\begin{array}{ccccc} 1 & 0.6935 & 0.0322 & 0.8028 & 0.1451\\ 0.6935 & 1 & -0.0185 & 0.7096 & 0.4532\\ 0.0322 & -0.0185 & 1 & 0.0118 & -0.0184\\ 0.8028 & 0.7096 & 0.0118 & 1 & -0.0155\\ 0.1451 & 0.4532 & -0.0184 & -0.0155 & 1 \end{array}\right]$$

High correlation (for example, between $x_{1}$ and $x_{4}$), medium correlation (for example, between $x_{2}$ and $x_{5}$) and low correlation (for example, between $x_{1}$ and $x_{3}$) can be found in this case. 

**Figure 2:**
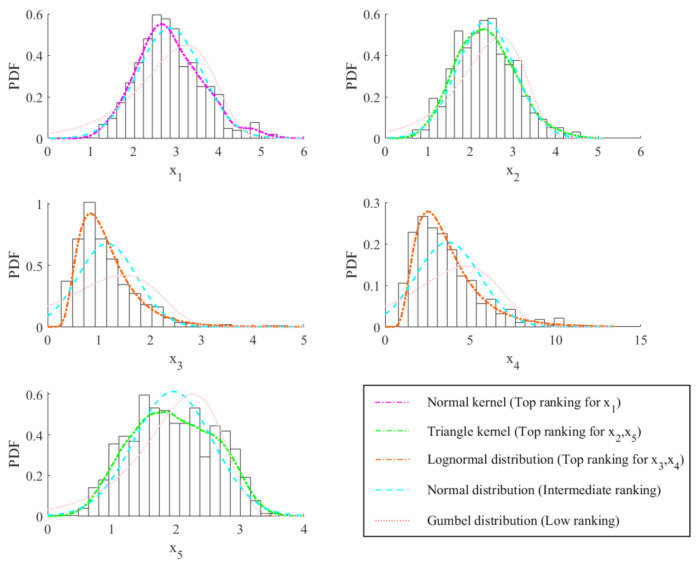
Univariate marginal PDFs of $X_{1}$ to $X_{5}$.

Table 2 shows the maximum log-likelihood value of different univariate marginal PDFs of $X_{1}$ to $X_{5}$. Using Equation (20), the optimal univariate marginal PDF of each dimension can be determined, and they are indicated by “_” (underline) in Table 2. The optimal PDFs of $X_{1}$ to $X_{5}$ are Normal kernel, Triangle kernel, Lognormal distribution, Lognormal distribution and Triangle kernel, respectively. In order to compare the fitting capacities of different PDFs shown in Table 2, Figure 2 shows the univariate marginal PDFs of $X_{1}$ to $X_{5}$. Each subplot shows the data histogram, the top ranking PDF (that is, the optimal marginal PDF in Table 2; line style as “dash-dot line"), an intermediate ranking PDF (that is, an intermediate ranking PDF in Table 2; line style as “dashed line") and a low ranking PDF (that is, a low ranking PDF in Table 2; line style as “dotted line"). From each subplot, it can be reconfirmed that the optimal marginal PDF of each dimension in Table 2 is the best model for uncertainty quantification of the corresponding component of X. It is worth noting that, from Table 2, even though X is a linear mapping of Z only composing very regular types of distributions described in Table 1, the optimal univariate marginal PDFs of X are not only from parametric but also from nonparametric models. This result shows that the introduction of both parametric and nonparametric models is necessary for breaking through the first limitation (i.e., insufficient types of probabilistic model candidates) described in Section 1 because it provides a large solution space for uncertainty quantification.

**Figure 3:**
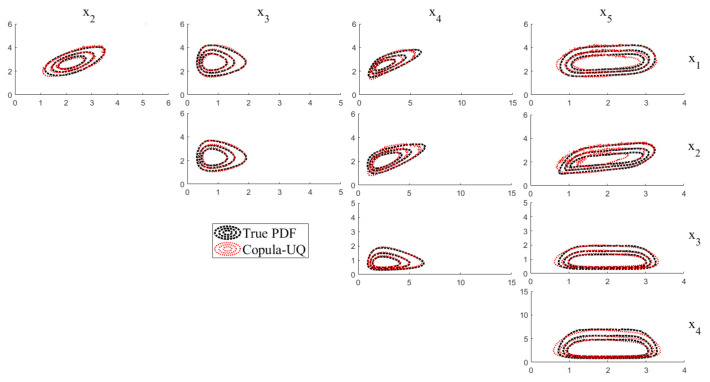
Projections of the multivariate joint PDF of $X_{1}$ to $X_{5}$.

The multivariate joint PDF of X is determined by Equation (11) with substituting the optimal marginal PDFs $\hat{Μ}$ along with the associated optimal parameters $\hat{\Theta }$ and the optimal parameter $\hat{\psi }$ of the multivariate Gaussian copula. Figure 3 shows the projections of the multivariate joint PDF of $X_{1}$ to $X_{5}$. Each subplot represents the projection of the multivariate joint PDF between two specific components of X. The black contour is the true PDF of Equation (25) while the green contour is the joint PDF by the proposed Copula-UQ. It can be shown that, even though the shape of the true PDF is irregular, the proposed Copula-UQ is capable of describing the statistical dependency structure. 

**Figure 4:**
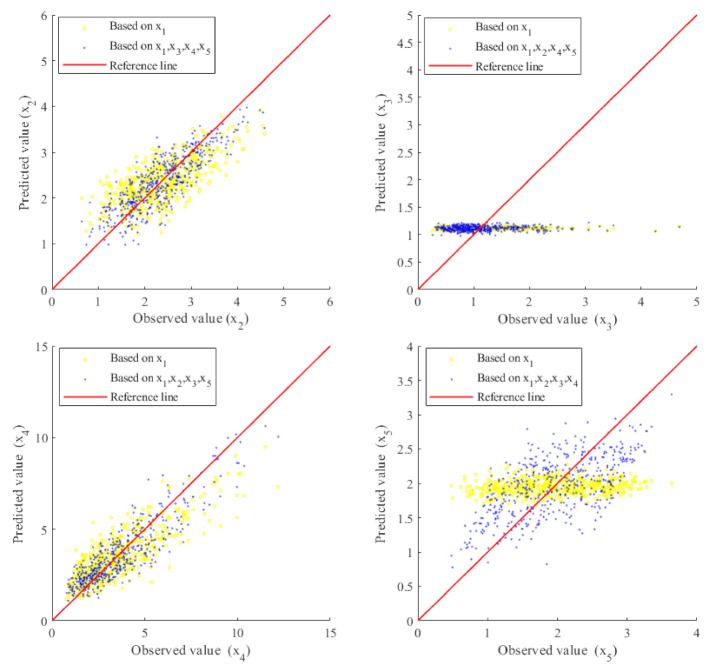
Comparisons of observed values and predicted values of $X_{2}$ to $X_{5}$.

Figure 4 shows the comparisons of observed values and predicted values of $X_{2}$ to $X_{5}$. The 45-degree reference line represents that the observed values and predicted values are identical. Each subplot shows the predicted value of the target variable, determined based on Equation (23), using the incomplete information (yellow dots) and complete information (blue dots) of the predictor variable. For example, for the yellow dots of the subplot in the upper left (for $x_{2}$), the target variable, observed predictor variable and unobserved predictor variable are $x_{\mathrm{ta}}\;=\;x_{2}$, $x_{o}\;=\;x_{1}$, $x_{\mathrm{uo}}=\left\{x_{3},x_{4},x_{5}\right\}$, respectively. For the blue dots of the subplot in the upper left (for $x_{2}$), $x_{\mathrm{ta}}=x_{2}$, $x_{o}=\left\{x_{1},x_{3},x_{4},x_{5}\right\}$, $x_{\mathrm{uo}}$ is an empty set. By comparing the scatter plots of yellow and blue dots, one can observe the evolution of the predicated value changes with respect to the amount of information given by the predictor variable. It can be anticipated that the predicted values can be improved (that is, the dots distributing closer to the 45-degree reference line) when given more information from the predictor variable. This conclusion can be confirmed from the subplots in the upper left (for $x_{2}$), lower left (for $x_{4}$) and lower right (for $x_{5}$) of Figure 4. Note that there is insignificant improvement of the predicted values of the subplots located in the upper right (for $x_{3}$), this is because of low correlations between $x_{3}$ and other components, shown in Equation (26). This result shows that the proposed formulation of Equation (23), breaking through the second limitation (i.e., negligence of probabilistic prediction using available information) by conducting computation of marginalization and conditioning, is capable of making predictions even though the information of the predictor variable is incomplete. 

### 4.2. Temperature Data of Multi-Sensor of a Monitored Bridge

Temperature is a critical loading factor for structures [32]. Variation of temperatures in structures significantly influences the material properties (for example, Young’s modulus [32]), static characteristics (for example, deflection and deformation [32]), dynamic characteristics (for example, structural frequencies [33,34,35], damping ratios and mode shapes [36]) and boundary conditions [37]. Temperatures, including ambient air temperature and structural component temperature, of a multi-sensor of a structure are uncertain due to the fact that they are affected by not only the ambient factors, including air temperature variation, solar radiation intensity, humidity and wind speed, but also the complex processes of heat transfer [38]. Practically, UQ in temperatures are conducted based on temperature data measured from multiple sensors installed in different locations of a monitored structure [38,39,40,41,42]. As these works utilized traditional PDF modelling approaches, and modelling of temperature-related random variables was limited to two-dimensional. Here, due to the capacity of the multivariate joint PDF modelling of the proposed Copula-UQ, the dimension can be extended to D-dimensional, where D is the number of temperature sensors selected in the analysis.

This study utilized the proposed Copula-UQ to analyze temperature data of the multi-sensor of the Dowling Hall Footbridge [36]. The bridge, located on the Medford campus of Tufts University, has a two-span continuous steel frame (each spam is 22 m) and a reinforced concrete deck. Temperatures of different locations are monitored using the type T thermocouples manufactured by Omega Engineering (measurement ranging from –250 to +350 °C). Multi-sensor layout for temperature monitoring can be referred to in Figure 7 of Reference [43]. There are in total ten temperature sensors and they can be divided into two sensor clusters according to their locations: the west span cluster and the east span cluster. The west span cluster includes sensors for pier temperature ($C_{1}$), bridge deck temperature ($C_{2}$), steel temperature at the south side ($S_{1}$), steel temperature at the north side ($S_{3}$) and air temperature ($A_{1}$). The east span cluster includes sensors for pier temperature ($C_{4}$), bridge deck temperature ($C_{3}$), steel temperature at the south side ($S_{2}$), steel temperature at the north side ($S_{4}$) and air temperature ($A_{2}$). 

**Figure 5:**
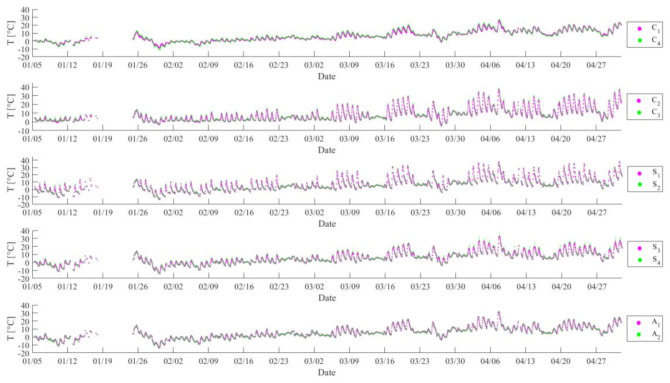
Time histories of ten temperature sensors beginning on January 5 2010 and ending on May 2 2010.

The temperature data can be accessed from Reference [44]. Figure 5 shows time histories of ten temperature sensors beginning on January 5 2010 and ending on May 2 2010. In each subplot, two sensors monitor the same type of temperature, but these two sensors belong to the west span cluster and the east span cluster, respectively. For example, in the first subplot, both $C_{1}$ and $C_{4}$ monitored pier temperature, but $C_{1}$ and $C_{4}$ belong to the west span cluster and the east span cluster, respectively. It can be observed that there is insignificant difference in measurement between two sensors monitoring the same type of temperature even though they belong to two different clusters. It is worth noting that there is difference between the steel temperature at the south and north sides of the bridge. The reason is due to the fact that the effects of sunlight to the south and north side are different. During the daytime hours, the sensor on the south side ($S_{1}$ and $S_{2}$) was significantly warmer than the sensor on the north side ($S_{3}$ and $S_{4}$) [36]. Therefore, temperature data of five sensors from the west span cluster ($C_{1}$, $C_{2}$, $S_{1}$, $S_{3}$, $A_{1}$) are utilized for UQ. The corresponding correlation coefficient matrix is:

(27) $$corr=\left[\begin{array}{ccccc} 1 & 0.9049 & 0.8698 & 0.9601 & 0.9691\\ 0.9049 & 1 & 0.8748 & 0.9345 & 0.9212\\ 0.8698 & 0.8748 & 1 & 0.9522 & 0.9357\\ 0.9601 & 0.9345 & 0.9522 & 1 & 0.9952\\ 0.9691 & 0.9212 & 0.9357 & 0.9952 & 1 \end{array}\right]$$

**Figure 6:**
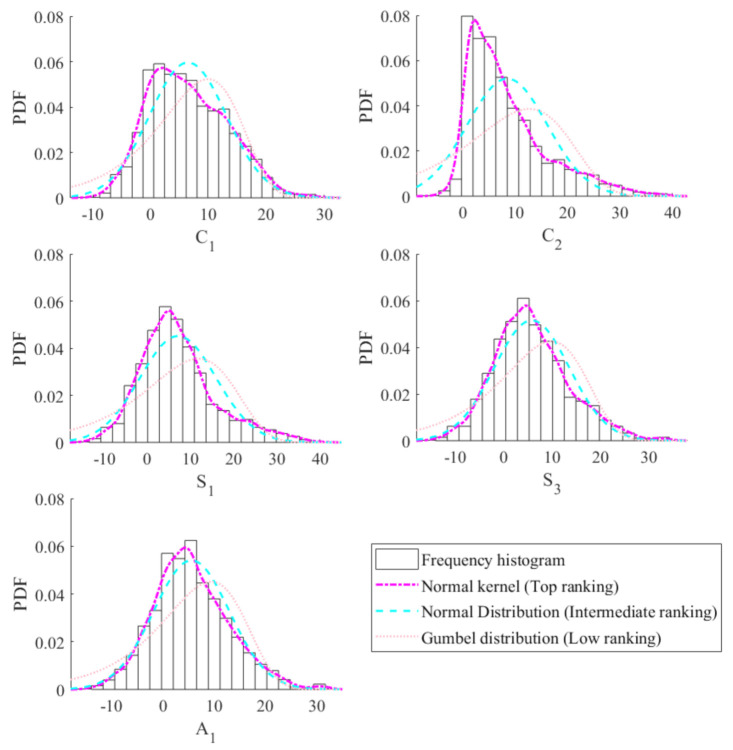
Univariate PDFs of $C_{1},C_{2},S_{1},S_{3}$ and $A_{1}$.

**Figure 7:**
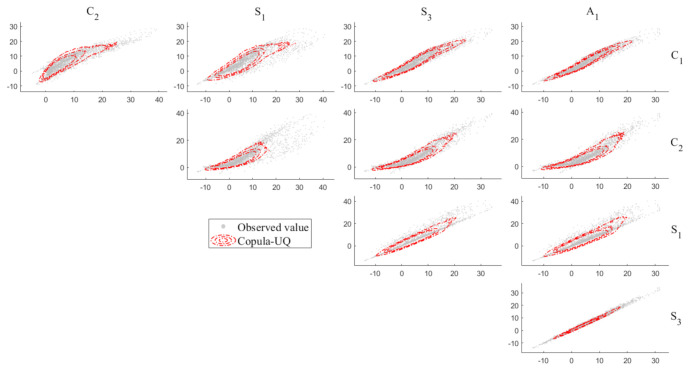
Projection of the multivariate joint PDF of $C_{1},C_{2},S_{1},S_{3}$ and $A_{1}$.

Table 3 shows the maximum log-likelihood value of the univariate marginal PDFs of $C_{1},C_{2},S_{1},S_{3}$ and $A_{1}$. It can be observed that the optimal PDFs of $C_{1},C_{2},S_{1},S_{3}$ and $A_{1}$ are distributed as the nonparametric model with the Normal kernel. Figure 6 shows the univariate marginal PDFs of $C_{1},C_{2},S_{1},S_{3}$ and $A_{1}$. It is obvious that in each subplot, the top-ranking model fits the frequency histogram better than the intermediate- and low-ranking models, reconfirming the model class selection results in Table 3. Figure 7 shows the projections of the multivariate joint PDF of $C_{1},C_{2},S_{1},S_{3}$ and $A_{1}$. It can be shown that the contours by the Copula-UQ are capable of quantifying the uncertainty of the multivariate temperature data.

**Figure 8:**
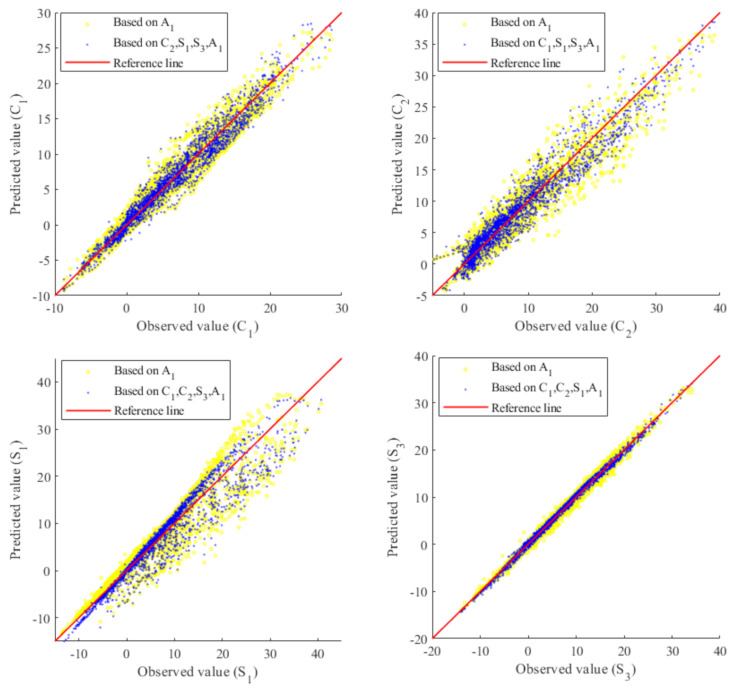
Comparisons of observed values and predicted values of $C_{1},C_{2},S_{1},S_{3}$ (training dataset: full monitoring dataset, test dataset: full monitoring dataset). ^2^ Note that the yellow dots correspond to incomplete information of the predictor variable due to missing data caused by a sensor fault issue.

Figure 8, in the same fashion as Figure 4, shows the comparisons of observed values and predicted values of $C_{1},C_{2},S_{1}$ and $S_{3}$ (training dataset: full monitoring dataset, test dataset: full monitoring dataset). Again, for each subplot, it can be observed that the predicted values can be improved when given more information of the predictor variable. Note that the yellow dots correspond to incomplete information of the predictor variable due to a sensor fault. For example, for the yellow dots of the subplot in the upper left (for $C_{1}$), the target variable, observed predictor variable and unobserved predictor variable are $x_{\mathrm{ta}}\;=\;C_{1}$, $x_{o}\;=\;A_{1}$, $x_{\mathrm{uo}}\;=\;\left\{C_{2},S_{1},S_{3}\right\}$, respectively. That is, the yellow dots show the predicted value of the target variable $C_{1}$ using the information from the observed variable of normal sensor $A_{1}$, but without using the information from the unobserved variable of fault sensors $C_{2}$, $S_{1}$, $S_{3}$ because of the fault status of these three sensors. For the blue dots of the subplot in the upper left (for $C_{1}$), $x_{\mathrm{ta}}\;=\;C_{1}$, $x_{o}\;=\;\left\{A_{1},C_{2},S_{1},S_{3}\right\}$, $x_{\mathrm{uo}}$ is an empty set. From the four subplots of Figure 8, although the sensor fault issue enlarges the fluctuation of the yellow dots, the proposed Copula-UQ gives satisfactory results as the available information of sensor $A_{1}$ is properly utilized for making predictions on the target variable. 

**Figure 9:**
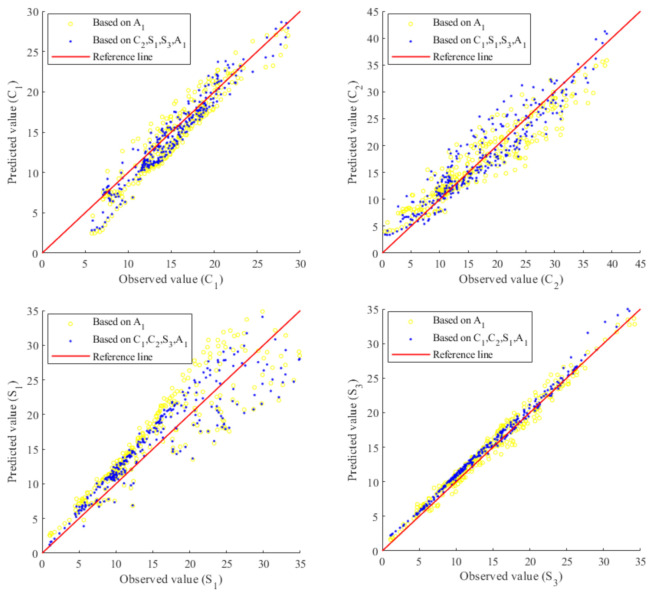
Comparisons of observed values and predicted values of $C_{1},C_{2},S_{1},S_{3}$ (training dataset: data covering first 90% days out of total monitoring period, test dataset: complement of training dataset). ^2^ Note that the yellow dots correspond to incomplete information of the predictor variable due to missing data caused by a sensor fault issue

For further validating the prediction capacity of the proposed Copula-UQ under data missing by sensor fault issue, a new computation is conducted as follows: (1) the monitored dataset was divided into the training dataset (data covering first 90% of days out of total monitoring period) and the test dataset (complement of the training dataset), (2) the marginal PDF along with the copula model was inferred based on the training dataset and (3) the prediction capacity of the trained copula model was validated based on the test dataset with or without data missing by sensor fault issue. Figure 9, in the same fashion as Figure 8, shows comparisons of observed values and predicted values of $C_{1},C_{2},S_{1}\;\mathrm{and}\;S_{3}$ (training dataset: dataset of first 90% of total number of the monitoring days, test dataset: complement of training dataset). It can be observed that the fluctuations of both the blue dots (corresponding to complete information from normal sensors) and the yellow dots (corresponding to incomplete information due to data missing by sensor fault issue) are acceptable. Therefore, it can be concluded that even though the training dataset is different from the test dataset, the proposed Copula-UQ still gives satisfactory results in multivariate PDF modelling and target variable prediction.

**Figure 10:**
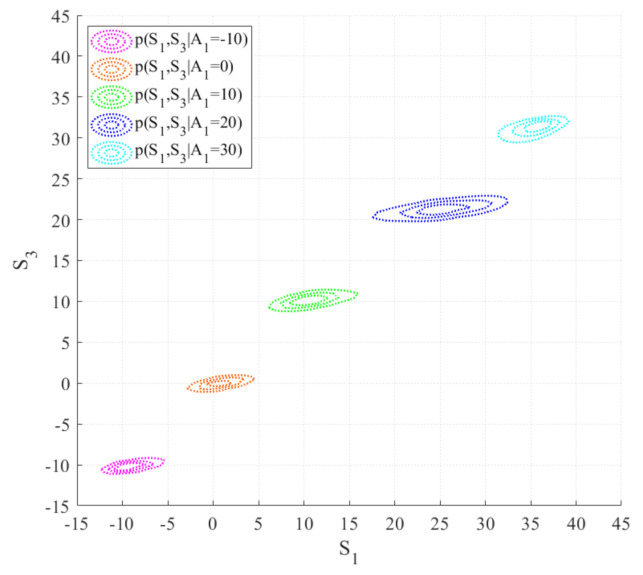
Predicted joint PDFs between $S_{1}$ and $S_{3}$ under incomplete information with given different values of $A_{1}$ only, and without observing information of fault sensors $C_{1}$ and $C_{2}$.

Figure 10 shows the predicted joint PDFs between $S_{1}$ and $S_{3}$ under incomplete information with different given values of $A_{1}$ only, and without observing information of fault sensors $C_{1}$ and $C_{2}$. That is, the joint PDF $p\left(S_{1},S_{3}|A_{1}\;=\;{\tilde{A}}_{1}\right)$ shows how the steel temperatures of the south and north sides evolve with changing the air temperature. As ${\tilde{A}}_{1}$ increases, the optimal values of $p\left(S_{1},S_{3}|A_{1}\;=\;{\tilde{A}}_{1}\right)$ increased accordingly. It can be observed that the differences between the steel temperature ($S_{1}$ or $S_{3}$) and the air temperature (${\tilde{A}}_{1}$) become more significant as ${\tilde{A}}_{1}$ increases. The reason is as follows: higher ${\tilde{A}}_{1}$ associates with higher solar radiation intensity. Given that the specific heat capacity of steel is higher than that of air, the increase of temperature of steel is more significant than that of air. It can also be observed that the temperature of $S_{3}$ is lower than that of $S_{1}$. This result coincides with the on-site situation of sunlight of the Dowling Hall Footbridge, in that the sunlight intensity to the north side ($S_{3}$) is lower than that to the south side ($S_{1}$) [36]. The predicted joint PDFs among the temperature of different locations of the structure are important pieces of information for uncertain thermal loading and can be utilized for thermal-induced structural response assessment. 

## 5. Conclusions

This paper proposed the Copula-UQ for multivariate joint PDF modelling, inference on model class selection and parameter identification, and probabilistic prediction using incomplete information, and presented one example using simulated data and one example using temperature data of a multi-sensor of a monitored bridge. For inference on univariate marginal PDFs, the results show that, in general cases, the optimal univariate marginal PDFs of different dimensions are different, so the introduction of both parametric and nonparametric models is necessary because it provides a large solution space for uncertainty quantification. For prediction on the target variable using the complete (from normal sensors) or incomplete information (due to missing data caused by a sensor fault issue) of the predictor variable, the proposed Copula-UQ is capable of obtaining the PDF of the target variable. The proposed methodology can be extended to tackle different multivariate joint PDF modelling problems in SHM with emphasizing the prediction purpose under incomplete information with a sensor fault issue. This important piece of information of the PDF of the target variable can be utilized for uncertainty propagation in further analysis. 

## Figures and Tables

**Table 1:** Probability density functions (PDFs) of $Z_{1}$ to $Z_{5}$ (Simulation data).

Random Variable	Distribution Type	PDF
$Z_{1}$	Normal	$f\left(z_{1}\right)=\frac{1}{\sqrt{2\pi }\sigma_{1}}e^{-\frac{{\left(z_{1}-\mu_{1}\right)}^{2}}{2\sigma_{1}{}^{2}}}$, $\mu_{1}=3,\;\sigma_{1}=1$
$Z_{2}$	Normal	$f\left(z_{2}\right)=\frac{1}{\sqrt{2\pi }\sigma_{2}}e^{-\frac{{\left(z_{2}-\mu_{2}\right)}^{2}}{2\sigma_{2}{}^{2}}}$, $\mu_{2}=2,\;\sigma_{2}=0.8$
$Z_{3}$	Lognormal	$f\left(z_{3}\right)=\left\{\begin{array}{cc} 0 & z_{3}<0\\ \frac{1}{\sqrt{2\pi }\sigma_{3}z_{3}}e^{-\frac{{\left(\ln z_{3}-\mu_{3}\right)}^{2}}{2\sigma_{3}{}^{2}}} & z_{3}\geq 0 \end{array}\right.$, $\mu_{3}=0,\;\sigma_{3}=0.5$
$Z_{4}$	Gamma	$f\left(z_{4}\right)=\left\{\begin{array}{cc} 0 & z_{4}\;\leq \;0\\ \frac{\beta_{4}{}^{\alpha_{4}}x^{\left(\alpha_{4}-1\right)}e^{-\beta_{4}z_{4}}}{\Gamma \left(\alpha_{4}\right)} & z_{4}>0 \end{array}\right.$, $\alpha_{4}\;=2,\beta_{4}=2$
$Z_{5}$	Uniform	$f\left(z_{5}\right)=\left\{\begin{array}{cc} 0 & z_{5}\leq a_{5},z_{5}\;\geq \;b_{5}\\ \frac{1}{b_{5}-a_{5}} & a_{5}<z_{5}<b_{5} \end{array}\right.$, $a_{5}=0.5,\;b_{5}=3.5$

**Table 2:** The maximum log-likelihood value of different univariate marginal PDFs of $X_{1}$ to $X_{5}$.

PDF	$X_{1}$	$X_{2}$	$X_{3}$	$X_{4}$	$X_{5}$
Normal kernel	−553.3514	−532.5992	−349.9818	−965.5745	−480.0222
Uniform kernel	−556.9401	−535.5691	−354.8022	−969.9313	−481.3604
Triangle kernel	−553.4045	−532.4293	−349.6769	−965.5385	−479.7110
Epanechnikov kernel	−555.2073	−534.0821	−351.9858	−967.8933	−480.6938
Normal distribution	−566.1014	−538.0549	−445.3173	−1045.1071	−494.6102
Lognormal distribution	−560.4108	−546.4166	−349.4094	−960.0402	−517.6582
Weibull distribution	−574.5840	−538.9670	−393.3779	−990.7425	−488.3424
Gamma distribution	−556.5564	−535.3848	−361.7671	−968.1071	−501.1078
Gumbel distribution	−638.5664	−600.1647	−623.2454	−1177.3065	−521.1552
Uniform distribution	−713.8654	−685.9265	−743.3989	−1217.4954	−574.5622

^1^ “_” indicates the optimal univariate marginal PDF.

**Table 3:** The maximum log-likelihood value of the univariate marginal PDFs of $C_{1},C_{2},S_{1},S_{3}$ and $A_{1}$.

PDF	$C_{1}$	$C_{2}$	$S_{1}$	$S_{3}$	$A_{1}$
Normal kernel	–8355.3340	–8216.0978	–8968.6926	–8737.8576	–8635.4290
Uniform kernel	−8359.5995	−8246.7759	−8979.0882	−8750.4474	−8645.4155
Triangle kernel	−8355.4031	−8222.3736	−8968.8041	−8738.2284	−8635.7345
Epanechnikov kernel	−8358.0363	−8232.9844	−8973.6371	−8743.8474	−8640.5933
Normal distribution	−8442.4746	−8790.1815	−9149.7283	−8813.6539	−8691.3643
Gumbel distribution	−8752.8354	−9405.0940	−9648.7378	−9218.5528	−9072.9188
Uniform distribution	−9210.6580	−9614.1435	−10,153.6737	−9855.9299	−9716.0754

^1^ “_” indicates the optimal marginal PDF.
